# Simultaneous Visualization of Enzymatic Activity in the Cytoplasm and at Polyphosphate Inclusions in *Beggiatoa* sp. Strain 35Flor Incubated with ^18^O-Labeled Water

**DOI:** 10.1128/mSphere.00489-18

**Published:** 2018-12-19

**Authors:** Simon Langer, Angela Vogts, Heide N. Schulz-Vogt

**Affiliations:** a Leibniz Institute for Baltic Sea Research Warnemünde (IOW), Rostock, Germany; University of Wyoming

**Keywords:** 18O-water, *Beggiatoa*, nanoSIMS, polyphosphate

## Abstract

Here we report on a new nanoscale secondary ion mass spectrometry (nanoSIMS) approach based on enzyme-mediated oxygen isotope exchange, which combines the visualization of general metabolic activity in the cytoplasm with insights into the activity of enzymes related to polyphosphate (polyP) inclusions. The polyP-accumulating strain of the large sulfur bacterium *Beggiatoa* was used as a model organism. *Beggiatoa* cultures were grown under oxic and anoxic conditions when exposed to either low- or high-sulfide conditions, which are known to influence polyP metabolism in this strain. Subsequent incubation with ^18^O-labeled water led to high ^18^O enrichments above the natural background in the cytoplasm and polyP granules derived from enzymatically mediated oxygen isotope exchange. The relative importance of polyP under the different sulfide regimes became evident by an apparent continued metabolic activity at polyP inclusions under stressfully high sulfide concentrations, in contrast to a decreased general metabolic activity in the cytoplasm. This finding confirms the role of polyP as a critical component in bacterial stress response and maintenance of a survival metabolism.

**IMPORTANCE** Microbial organisms exert a large influence on the environment as they directly affect the turnover of essential elements. This is particularly true for polyphosphate-accumulating large sulfur bacteria, which can either accumulate phosphate as polyphosphate or degrade it and release phosphate into the environment, depending on environmental conditions. This study presents a new approach to simultaneously visualize general metabolic activity and enzymatic activity at polyphosphate granules by incubation with ^18^O-labeled water as the only stable isotope tracer. For this purpose, the well-studied *Beggiatoa* sp. strain 35Flor was used as a model organism and was exposed to different stress regimes. General metabolic activity was strongly impaired during high-stress regimes. In contrast, intense intracellular polyP cycling was not restricted to favorable or stressful conditions, highlighting the importance of polyP for general cell physiology, especially during hostile conditions. The nanoSIMS approach adds a new tool to study microorganisms involved in phosphorus cycling in the environment together with the identification of general metabolic activity.

## Abstract (precis)

Microbial organisms exert a large influence on the environment as they directly affect the turnover of essential elements. This is particularly true for polyphosphate-accumulating large sulfur bacteria, which can either accumulate phosphate as polyphosphate or degrade it and release phosphate into the environment, depending on environmental conditions. This study presents a new approach to simultaneously visualize general metabolic activity and enzymatic activity at polyphosphate granules by incubation with ^18^O-labeled water as the only stable isotope tracer. For this purpose, the well-studied *Beggiatoa* sp. strain 35Flor was used as a model organism and was exposed to different stress regimes. General metabolic activity was strongly impaired during high-stress regimes. In contrast, intense intracellular polyP cycling was not restricted to favorable or stressful conditions, highlighting the importance of polyP for general cell physiology, especially during hostile conditions. The nanoSIMS approach adds a new tool to study microorganisms involved in phosphorus cycling in the environment together with the identification of general metabolic activity.

## OBSERVATION

The identification and localization of bacterial activity are major goals in environmental microbiology. One method often used is the application of stable isotopes, which enables cell-specific labeling of metabolic active microorganisms in combination with secondary ion mass spectrometry (SIMS) (1). This is mostly conducted with isotopes of nitrogen (N) or carbon (C) but is not feasible for phosphorus (P)-bearing molecules due to the presence of only one stable P isotope. Instead, stable oxygen (O) isotope ratios in phosphate (δ^18^O_p_) have been used regularly in recent years to obtain insights into extra- and intracellular P cycling (2, 3). Signals of δ^18^O_p_ are often dominated by the ubiquitous activity of intracellular pyrophosphatases (PPases), which catalyze a full oxygen isotope exchange between oxygen-water and oxygen-phosphate within minutes (4–6). This opens the possibility to label oxygen-rich molecules, like polyphosphate (polyP), with introduced ^18^O-enriched water, if they are metabolically used.

P and energy-rich polyP can consist of up to 1,000 phosphate residues linked by high energetic phosphoanhydride bonds (7). PolyP-accumulating microorganisms, like most members of the genus *Beggiatoa,* have the potential to store large amounts of polyP in response to environmental conditions (8, 9). Laboratory studies showed that the well-studied *Beggiatoa* sp. strain 35Flor synthesizes polyP during favorable oxic growth conditions, while polyP is degraded during hostile, anoxic, and highly sulfidic conditions leading to phosphate (P_i_) release into the environment (9).

As the polyP metabolism of these large bacteria is well-known, they were used in this study as a model organism to investigate enzymatically mediated oxygen isotope exchange reactions leading to ^18^O enrichments. Enrichments in polyP due to the activity of polyP-related enzymes, and in the cytoplasm, most likely related to enzyme-mediated oxygen isotope exchange at amino acids forming proteins (10, 11), were visualized with nanoscale secondary ion mass spectrometry (nanoSIMS). Filaments were grown in test tubes with opposing gradients of oxygen in the top agar and sulfide in the bottom agar. Incubations with ^18^O-labeled water were performed with oxic, anoxic, and cooled control treatments, being exposed to either high or low sulfide fluxes.

### Distribution of enzyme-mediated ^18^O enrichments within cells.

**FIG 1:**
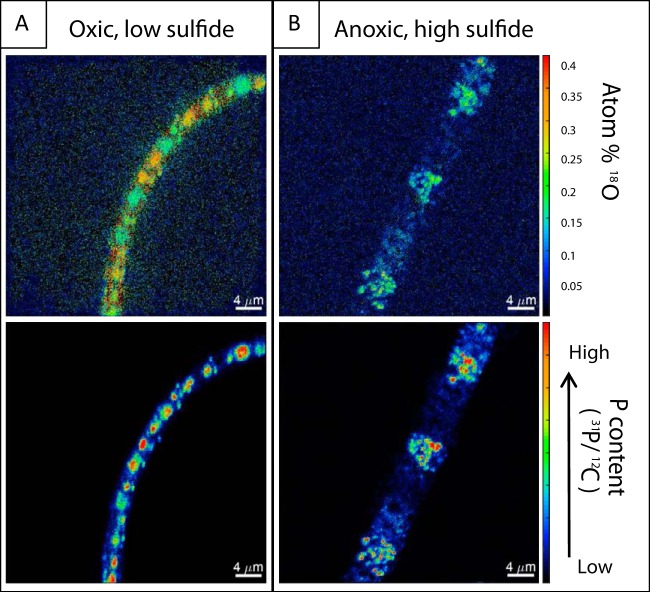
The distributions of ^18^O and P are shown for the physiological most distinguishable incubation conditions. (A) Optimal growth condition (oxic, low sulfide); (B) stressful condition (anoxic, high sulfide flux). ^18^O enrichments in atom% are presented in the top panels, and phosphorus content normalized to carbon is present in the bottom panels. The widths of filaments differ between the treatments caused by more extensive sulfur accumulation under elevated sulfide fluxes (25).

**FIG 2:**
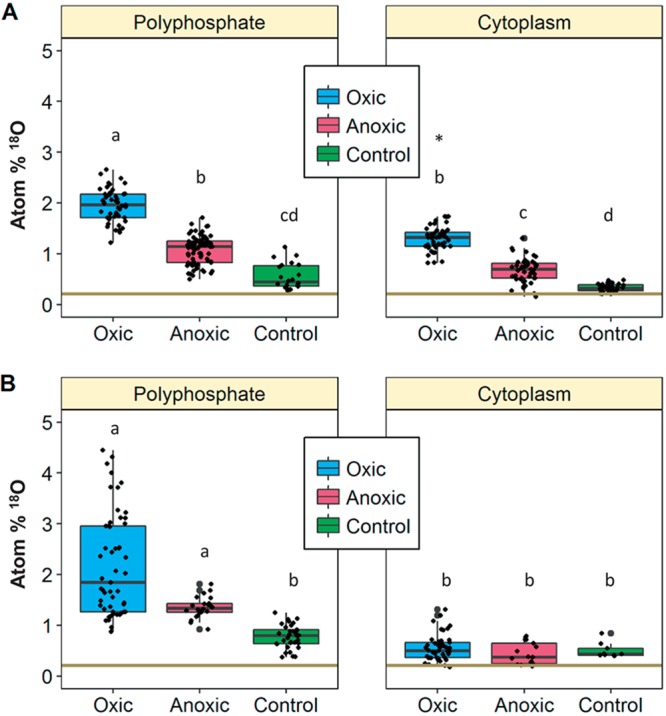
Boxplots from ROIs based on P counts for different treatments (oxic, anoxic, and control) during low sulfide flux (A) and high sulfide flux (B). Different letters indicate boxes that differ significantly between treatments exposed to the same sulfide fluxes (*P* < 0.05). Boxes with the same letters are not significantly different (*P* > 0.05). The asterisk indicates that the box was significantly different between different sulfide concentrations within the same treatment. The horizontal line at 0.2 atom % presents the natural abundance of ^18^O.

In order to differentiate between polyP granules and the cytoplasmic background, regions of interests (ROIs) were defined on the basis of P content (details in “NanoSIMS analyses” below). In all treatments, polyP granules were highly enriched in ^18^O compared to the natural background of 0.2 atom % ^18^O (12) (Fig. 1 and Fig. 2). Since the P-O bonds in polyP are resistant to inorganic hydrolysis (13), the observed oxygen isotope exchange between ^18^O-labeled water and polyP indicates enzymatic activity (14, 15). ^18^O enrichments in polyP are supposed to originate from the addition of already ^18^O-labeled phosphate after previous oxygen isotope exchange mediated by PPases (4–6). Alternatively, oxygen isotope exchange can occur during cleavage of the terminal phosphate residue in polyP granules by exopolyphosphatases with concurrent oxygen isotope exchange being associated with phosphatases (e.g., 16–18).

In contrast to polyP granules, ^18^O in the cytoplasm showed divergent degrees of enrichment depending on the incubation conditions (Fig. 1 and Fig. 2). Filaments exposed to low sulfide fluxes under oxic conditions were enriched in ^18^O in the whole cytoplasm, whereas much less enrichment in the cytoplasm was observed under high sulfide fluxes and anoxic incubation conditions. Enrichment in the cytoplasm is most likely allocated predominantly in proteins, which constitute up to 55% of the cell weight (dry weight) (19), and thus, serves as a biomarker for metabolic activity (20). The low enrichments under stressfully high sulfide conditions indicate a general reduction of standard physiological activity such as protein synthesis leading to less intense ^18^O exchange. Even lower ^18^O enrichments were present in the control (Fig. 2), which was incubated under cooled conditions to reduce the general enzymatic activity (see “H_2_^18^O incubation of *Beggiatoa* sp. strain 35Flor” below), confirming that biological activity is predominantly responsible for ^18^O enrichments in the filaments. Thus, isotope exchange with ^18^O-labeled water proved to be a suitable tracer for the distribution of enzymatic activity within filaments, especially in the case of polyP granules.

### PolyP as a last resort under environmental stress.

Figure 2 shows ^18^O enrichments in polyP granules and the cytoplasm after incubation with low (Fig. 2A) and high sulfide fluxes (Fig. 2B). Under low sulfide fluxes, both polyphosphate inclusions and the cytoplasm showed the highest ^18^O enrichments under oxic conditions and lower values under anoxic conditions. Nevertheless, under both conditions, the enrichments at polyP inclusions and in the cytoplasm were significantly higher than in the control treatment. The ^18^O-enriched cytoplasm during both oxic and anoxic incubation conditions indicates ongoing standard metabolic processes. Thus, even under this energetically unfavorable condition, a general metabolism of the cell at a lower level could be maintained. In contrast to low sulfide treatments, ^18^O enrichments in the cytoplasm under high sulfide fluxes were at low levels similar to those for the control treatment, both in oxic and anoxic treatments (Fig. 2). This indicates that under high sulfide fluxes, basic metabolic processes within the cytoplasm were strongly impaired. However, the enzymatic activity located at polyP inclusions remained at a high level, similar to those observed in the low-sulfide treatment (Fig. 2B). This restriction of metabolic activity at polyP granules, which is induced by high sulfide fluxes, is further supported by comparing the relative ^18^O enrichments between polyP and the cytoplasm in the different treatments (Table 1). Relative enrichments were distinctly higher during unfavorably high sulfide fluxes. In contrast to this, ratios at low sulfide fluxes were comparable to those of the control. The restriction of metabolic activity at polyP granules indicates a pivotal role of polyP during environmental stress conditions. Here, it is induced by elevated sulfide concentrations, but it is probably also important under nutrient-limited (21) and energy-limited (22) environmental conditions.

**TABLE 1:** Mean values of ^18^O enrichments in ROIs defined as polyP and as cytoplasm and the ratio between the means of enrichments in polyP and cytoplasm^*a*^

Region or parameter	Mean ^18^O enrichments (atom %)					
	Oxic, high H_2_S	Oxic, low H_2_S	Anoxic, high H_2_S	Anoxic, low H_2_S	Control, high H_2_S	Control, low H_2_S
ROIs defined as polyP	2.2	2.0	1.4	1.1	0.8	0.5
	SD = 1.0	SD = 0.3	SD = 0.2	SD = 0.3	SD = 0.2	SD = 0.2
	*n* = 53	*n* = 48	*n* = 22	*n* = 78	*n* = 36	*n* = 23

ROIs defined as cytoplasm	0.6	1.3	0.4	0.7	0.5	0.3
	SD = 0.2	SD = 0.2	SD = 0.2	SD = 0.2	SD = 0.2	SD = 0.1
	*n* = 44	*n* = 44	*n* = 17	*n* = 45	*n* = 10	*n* = 29

**PolyP/cytoplasm** ** ratio**	**3.9**	**1.5**	**3.0**	**1.6**	**1.6**	**1.4**

a It becomes evident that the polyP/cytoplasm ratio is distinctively higher in treatments exposed to high sulfide fluxes, both under oxic and under anoxic conditions. The polyP/cytoplasm ratio and values are shown in boldface type.

### PolyP and intracellular P cycling.

Low sulfide concentrations led to significantly higher ^18^O-enriched PolyP granules in the oxic incubation than in the anoxic treatment (Fig. 2). A pronounced higher enrichment under oxic conditions was also present in the high sulfide treatment, but due to the large scatter in the strength of ^18^O enrichments, this difference is not significant (Fig. 2). Since phosphate in the surrounding medium was depleted after 7 days of growth (9) (see “H_2_^18^O incubation of *Beggiatoa* sp. strain 35Flor” below), ^18^O enrichments at polyP granules could not originate from external phosphate in the medium being enriched by the activity of PPase. Instead, ^18^O enrichments indicate enzymatic activity originating from an intense intracellular recycling of phosphate, which is presumably mediated by polyphosphate kinases. These enzymes catalyze the reversible formation of polyP from ATP (23, 24) and connect the high energetic phosphorus pools of ATP and polyP. Apparently, the internal polyP pool of the analyzed cells was highly mobile and enzymatically used also under oxic conditions and not predominantly when exposed to anoxic, sulfidic conditions as formerly assumed for *Beggiatoa* and other large sulfur bacteria (8, 9). The high enzymatic activity at polyP granules during oxic, favorable conditions underlines the important role of polyP for the overall cell metabolism. To summarize, ^18^O enrichments indicate no significant differences in polyP utilization between stressful (high-sulfide) and less-stressful (low-sulfide) conditions and show high activity also during oxic incubation conditions. Thus, the enzymatic activity at polyP inclusions indicated by ^18^O enrichments is always present regardless of the environmental redox conditions but becomes crucial when the filaments are exposed to sulfidic stress.

### Conclusion.

The nanoSIMS approach enables the concurrent visualization of general metabolic activity and enzymes related to polyP metabolism. The activity of polyP-related enzymes was demonstrated under both favorable and stressful conditions and underlines the importance of polyP for cellular metabolism, especially when exposed to high sulfide fluxes. The method presented here expands the application of stable isotope tracers for microbiological studies involving P-O-containing molecules and opens new possibilities to study the relative importance of polyP-associated metabolism in environmental samples.

### H_2_^18^O incubation of *Beggiatoa* sp. strain 35Flor.

*Beggiatoa* sp. strain 35Flor was cultivated in opposing gradients of oxygen and sulfide as described elsewhere in detail (25), with reduced phosphate concentrations of 20 µM leading to phosphate depletion within the mat after 7 days of growth (9). Sulfide concentrations in the bottom agar were 4 mM (low sulfide) and 24 mM (high sulfide).

Seven days after inoculation of the medium, ^18^O- water (97 atom % ^18^O; Sigma-Aldrich) was added to the mat of *Beggiatoa* filaments at the oxic/anoxic interface. Oxic treatments were incubated under continued microaerobic (oxic) conditions; anoxic incubation conditions were established by thoroughly replacing the atmosphere above the agar with N_2_. Control incubations were transferred to 4°C after the addition of ^18^O-labeled water. Oxic and anoxic incubations were performed with both high- and low-sulfide concentrations in the bottom agar. The control incubation was conducted under oxic conditions with low-sulfide concentration in the bottom agar. ^18^O-water incubations proceeded for 24 h, before single filaments were picked and transferred onto polycarbonate filters (PC), which were stored at −24°C until further processing.

### NanoSIMS analyses.

PC filters were thawed at room temperature and incubated with DAPI for at least 30 min. Subsequently, cell integrity was visually confirmed by fluorescence microscopy using a LMD 6500 (Leica, Wetzlar, Germany; filter cube B/G/R). The positions of agar free filaments were marked with a laser pulse, which facilitated orientation for nanoSIMS analyses. The samples were coated with ca. 30 nm gold with a Cressington 108auto sputter coater (Watford, United Kingdom). At the marked position, SIMS imaging was performed using a NanoSIMS 50L instrument (Cameca, Paris, France) at the Leibniz-Institute for Baltic Sea Research Warnemünde (IOW). A ^133^Cs^+^ primary ion beam was used to erode and ionize atoms of the sample. Images of secondary electrons, ^12^C^−^, ^16^O^−^, ^18^O^−^, and ^31^P^−^, were recorded simultaneously using mass detectors equipped with electron multipliers (Hamamatsu) for the ions. The mass resolving power was adjusted to be sufficient to suppress interferences at all masses allowing, e.g., the separation of ^13^C^−^ from interfering ions such as ^12^C^1^H^−^. Prior to the analysis, sample areas of 50 by 50 µm were sputtered for 2 min with 600 pA to erode the gold, clean the surface, and reach the steady state of secondary ion formation. The primary ion beam current during the analysis was 1 pA; the scanning parameters were 512 by 512 pixels for areas of 45 by 45 µm, with a dwell time of 250 µs per pixel. Sixty planes were analyzed.

Data analysis was performed with the Look@NanoSIMS software (26). Previous to plane accumulation and drift correction, a maximum of three erroneous planes (with e.g., signal instabilities) were removed. Regions of interest (ROIs) were determined based on P counts normalized to C counts (P/C). P rich (reddish spots) in contrast to P poor (bluish spots) were manually assigned as ROIs (see Fig. S1 in the supplemental material). Counts for each individual ROI were obtained for ^12^C^−^, ^31^P^−^, ^18^O^−^, and ^16^O^−^, which were used to calculate C/P ratios and ^18^O enrichments in atom %. ^18^O enrichments were plotted against C/P (relative phosphorus content) to determine the threshold value between ROIs representing polyP and ROIs representing cytoplasm (Fig. S2). The cutoff was set at a C/P ratio of 18 with ratios below 18 regarded as polyP (relatively high P content) in contrast to ratios above 18 (relatively low P content). This was based on the most pronounced gap in most treatments between adjacent ROIs (Fig. S2). Significant differences between ROIs assigned as polyP versus ROIs assigned as cytoplasm based on C/P ratios were confirmed by statistical analyses (details below). However, it is important to note that 18 is the best-suited value for these measurements, but it is not valid as a general cutoff. Since nanoSIMS does not give absolute values, individual cutoffs have to be defined for each individual study.

10.1128/mSphere.00489-18.1 FIG S1 Two example ROIs for both polyP (yellow regions) and cytoplasm (white regions), defined by manual assignment, shown as overlays on the data from Fig. 1. Download FIG S1, EPS file, 3.4 MB. Copyright © 2018 Langer et al. 2018 Langer et al. This content is distributed under the terms of the Creative Commons Attribution 4.0 International license.

10.1128/mSphere.00489-18.2 FIG S2 C/P ratios, indicative of P content, plotted against ^18^O enrichments for all determined ROIs. Decreased C/P ratios (high P content) are apparent in areas defined as polyP compared to regions defined as cytoplasm, especially under high sulfide conditions. Download FIG S2, TIF file, 1.4 MB. Copyright © 2018 Langer et al. 2018 Langer et al. This content is distributed under the terms of the Creative Commons Attribution 4.0 International license.

### Statistical analysis.

The software “R” was used for statistical analyses. ROIs from filaments within the same treatment were pooled after confirming no significant differences between ROIs of filaments incubated under the same conditions based on P, ^16^O, and ^18^O counts. Significant differences in C/P ratios (indicative for P content) between ROIs defined as polyP and ROIs defined as cytoplasm were confirmed with the Whitney U-test. The nonparametric Kruskal-Wallis test followed by the Dunn’s test were performed to identify differences in ^18^O enrichments between the treatments and between polyP granules and the cytoplasm.
