# Supplementary figures and images for: Simultaneous Visualization of Enzymatic Activity in the Cytoplasm and at Polyphosphate Inclusions in Beggiatoa sp. Strain 35Flor Incubated with 18O-Labeled Water
Source: mSphere. 2018 Dec 19;3(6):e00489-18. doi: 10.1128/mSphere.00489-18 (PMC6300685; doi:10.1128/mSphere.00489-18)

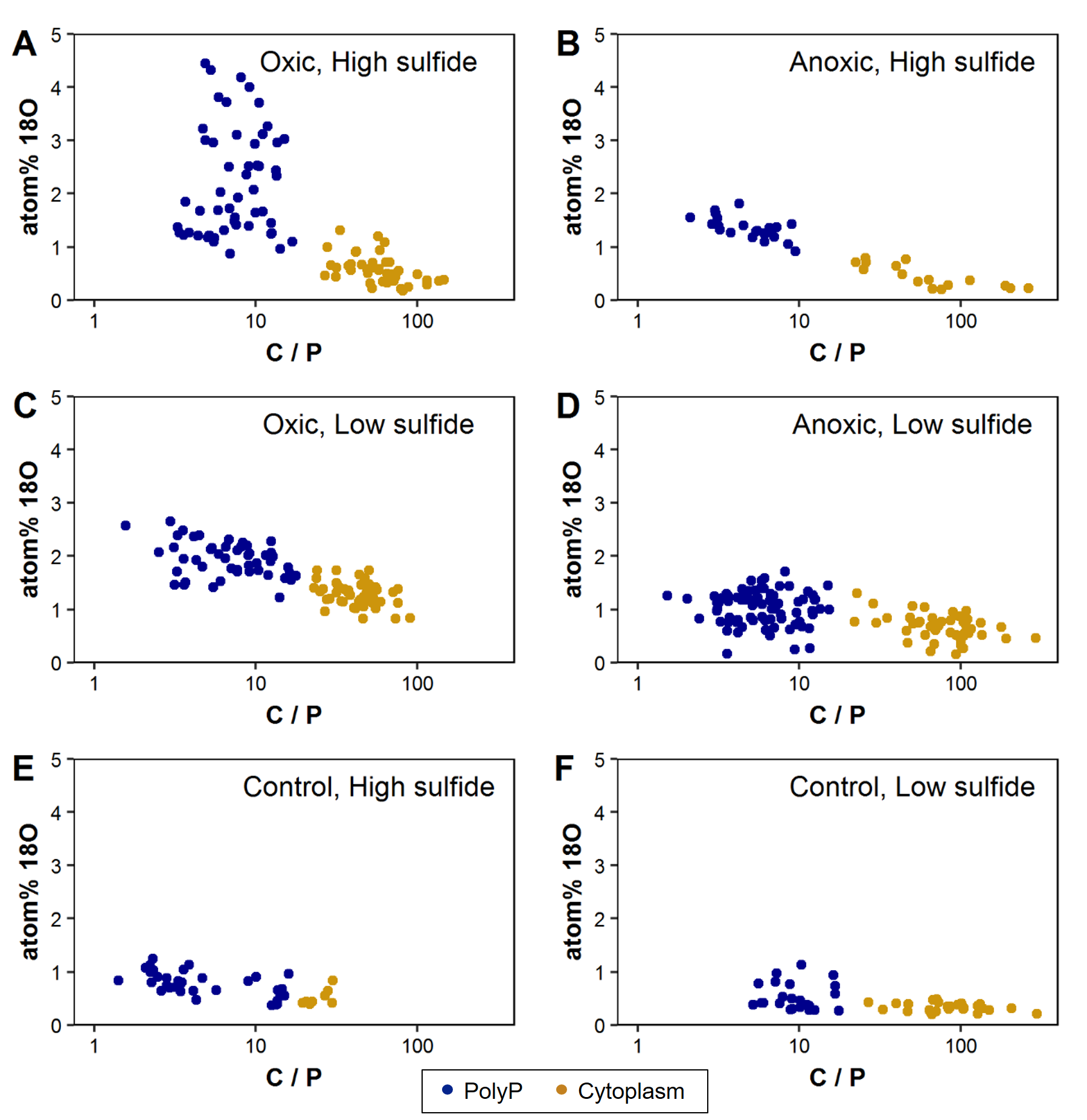

Supplement: FIG S2 [file sph006182726sf2.tif]
